# Supplementary material for: Guaranteed minimum withdrawal benefits with high-water mark fee structure
Source: PLoS One. 2024 May 21;19(5):e0302740. doi: 10.1371/journal.pone.0302740 (PMC11108128; doi:10.1371/journal.pone.0302740)
Supplement: S1 File — (ZIP) [file pone.0302740.s002.zip › Highwatermark (jump regime switch)/OSAmeetings.pdf]

Guaranteed minimum withdrawal benefits with high-water mark fee structure

Yichen Han[1], Lianxia Wu[2*], Dongchen Li[1] and Jiaqi Han[3]

[1] School of Statistics, East China Normal University, Shanghai 200241, China; 52214404004@stu.ecnu.edu.cn

[2] Population Research Institute in School of Social Development, East China Normal University, Shanghai 200241, China

[3] School of Mathematical Sciences, Fudan University, Shanghai 200433, China

[*] Correspondence: lxwu@soci.ecnu.edu.cn; Tel: +86-173-1787-9687

*Funding: This work is supported by National Social Science Fund in China (19CRK010)

Conflict of interest: The authors declare that they have no conflict of interest.

Abstract: The Guaranteed Minimum Withdrawal Benefit (GMWB), an adjunct incorporated within variable annuities, commits to reimbursing the entire initial investment regardless of the performance of the underlying funds. While extensive research exists in financial and actuarial literature regarding the modeling and valuation techniques of GMWBs, much of it is founded on a static fee structure. Our study introduces an innovative fee structure based on the high-water mark (HWM) principle for the pricing of GMWBs, employing numerical solutions through the Monte Carlo method for solving the stochastic differential equation (SDE). Furthermore, a companion piece of research addresses the risk management of GMWBs within the same analytical framework as the pricing component, an aspect that has received limited attention in the existing literature. In assessing the necessary capital reserves for unforeseen losses, our methodology involves the computation of two risk metrics associated with the tail distribution of net liability from the insurer's perspective, Value-at-Risk (VaR) and Conditional-Tail-Expectation (CTE). Comprehensive numerical results and sensitivity analyses are also provided.

Keywords: Guaranteed minimum withdrawal benefit; Variable annuity; High-water mark fee structure; Risk-neutral valuation; Risk measure; Monte Carlo simulation

1. Introduction

Variable annuities (VAs) represent life insurance agreements established between a policyholder (PH) and an insurance company. As an initiation of a typical VA contract, the policyholder provides a lump-sum premium payment to the insurer. Subsequently, the premium is invested in the mutual funds that mirror the performance of a reference portfolio, thereby enabling the PH to partake in the equity markets with the insurer's commitment to disbursing variable periodic payments on specified future dates along with stable long-term

embedded guarantees. To finance these guarantees, the insurer periodically diminishes the investment account by levying insurance fees. These embedded guarantees encompass a variety of types, including guaranteed minimum death benefit (GMDB), guaranteed minimum accumulation benefit (GMAB), guaranteed minimum income benefit(GMIB), guaranteed lifetime withdrawal benefits (GLWB), and guaranteed minimum withdrawal benefits (GMWB). Notably, the last four mentioned can be categorized collectively as Guaranteed Minimum Living Benefits (GMLB). There exists a body of review literature that delves into the relationships and valuation issues of these guarantees, as evidenced by references [1–3]. Additionally, their practical applications have been explored in literature[4–7].

In recent years, variable annuities featuring GMWB have garnered considerable attention. The GMWB provision enables policyholders to make fund withdrawals at each time interval, ensuring the complete return of the initial purchase payment over the policy's lifespan, even if the account balance diminishes to zero before reaching maturity. In the event the account maintains a positive balance at maturity, the remaining funds are disbursed to the policyholder. A predetermined contractual withdrawal rate is specified, allowing policyholders to make withdrawals at or below this rate without incurring penalties. These periodic withdrawals, along with insurance fees, lead to a depletion of the investment account.

The modeling and pricing of GMWB have been extensively explored in the fields of finance and actuarial literature. Notably, Milevsky and Salisbury [8], introduced a valuation model for variable annuities featuring GMWB, encompassing both static and dynamic modeling frameworks. Feng and Volkmer [9] were pioneers in providing analytical solutions to the pricing problem of GMWB, considering both the policyholder's and insurer's perspectives. Peng et al. [10] derived analytic approximation solutions for the fair value of GMWB under the Vasicek stochastic interest rate framework, offering lower and upper bounds on the price functions. Dai et al. [11] developed a unique stochastic control model to price GMWB, taking into account policyholder behavior. Kling et al. [12] investigated the influence of stochastic volatility on the pricing and hedging of GMWB. Goudenège et al. [13] conducted an investigation into the computation of value and Greeks for a GMWB variable annuity, considering the coexistence of stochastic volatility and stochastic interest rates within the Heston–Hull–White model. Fontana and Rotondi [14] conducted an extensive analysis regarding the valuation of a broad category of GMWB annuities. Their work factored in key features like step-up provisions, bonus options, surrender capabilities, and notably, considerations for mortality risk and death benefits.

While various techniques and structures exist for GMWB policies, a predominant approach employs a static fee structure. This structure entails fees that are proportionate to the present value of the investment account, with the proportion being fixed. The persistence of this static fee structure may have contributed to a decline in sales within the VA market in recent years. Therefore, it is imperative to propose and analyze straightforward alterations in fee structures (see Bernard and Moenig[15]). In addition to the conventional fee structure previously discussed, several alternative fee structures also have been explored. For instance, Bernard et al. [16] argued that the constant fee structure could lead to a misalignment of risk and income, particularly with increasing surrender risk. To address this, they introduced a state-dependent fee structure, imposing a constant fee only when the investment account value falls below a specified threshold. Cui et al. [17] theoretically analyzed the benefits of a VIX-linked fee structure under a parametric model, demonstrating that it reduces the insurer's liability sensitivity to market volatility. Bernard and Moenig [15] proposed a dynamic, time-dependent fee rate to alleviate the challenge of high fees without compromising the insurer's profit. Under this proposal, the insurance fee decreases only after a certain time threshold. Landriault et al.[18] were pioneers in applying the novel high-mark fee structure

to variable annuities embedded with GMDB and GMMB. Wang and Zou [19] delved into the intricate design of fee structures for variable annuities, framing it as a stochastic control problem. In their study, the insurer was granted the flexibility to choose a fee structure satisfying specified budget constraints. The notable finding was that the optimal fee structure took on a barrier-type form with a time-dependent free boundary, assuming a no-surrender scenario. On the other hand, Kirkby and Aguilar [20] investigated the valuation and optimal surrender dynamics of variable (equity-linked) annuities within a Lévy-driven equity market framework. Their analysis incorporated mortality risk and introduced a practical periodic fee structure that could dynamically vary over time, levied as a proportion of the fund value.

However, the literature on the risk management of variable annuity guarantees remains limited. Feng and Volkmer[9] were the first to analytically calculate risk measures for variable annuity guaranteed benefits, with a focus on GMDB and GMDB as illustrative examples. Feng [17] extended this analytical approach to cover GLWB and GMWB, respectively. Additionally, Feng and Yi [21] delved into the exploration of risk measures and hedging strategies for Guaranteed Minimum Accumulation Benefit (GMAB) within the same analytical framework. Godin et al. [22] introduced a flexible risk decomposition method for life insurance contracts, incorporating multiple risk factors and providing numerical examples that analyze the relative impact of equity, interest rate, and mortality risk for GMMB policies, thus contributing to our understanding of this domain.

This study investigates the impact of the high-water mark fee structure on the Guaranteed Minimum Withdrawal Benefit (GMWB) from three distinct perspectives. Firstly, we establish the pricing model for GMWB under the high-water mark fee structure. Serving as an extension of both the constant fee structure and the state-dependent fee structure, the high-water mark (HWM) fee structure presents several advantageous attributes. Secondly, building upon the findings from the initial segment, we compute two specific risk measures associated with the net liability distribution to effectively manage risk, employing a reversibility principle. Lastly, we analyze policyholders' surrender behaviors. During the policy term, the policyholder retains the option to surrender the Variable Annuities contract before reaching maturity, albeit subject to predefined surrender penalties. This penalty framework aims to deter early surrenders effectively. In the latter part of this paper, we ascertain the optimal surrender strategy, providing valuable insights into pricing policy value using the Least Square Monte Carlo simulation.

The paper is structured as follows. In the subsequent section, we employ a risk-neutral pricing approach to ascertain equitable insurance fees for the Guaranteed Minimum Withdrawal Benefit with the high-water mark fee structure. This analysis is conducted from the insurer's perspective and is situated within the same analytical framework as Feng and Volkmer (2016). In Section 3, we continue our examination from the insurer's standpoint, focusing on the computation of tail probabilities and expectations about the net liability, as defined in Feng and Volkmer (2012). This computation aids in determining the necessary capital reserves to mitigate potential losses. We present numerical examples that illustrate the fair valuation of insurance fees and the computation of risk measures under the high-water mark fee structure. In Section 4, we provide the numerical outcomes for the preceding section. Additionally, we delve into the exploration of optimal surrender timing and its correlation with policy value through the implementation of the Least Square Monte Carlo simulation. To facilitate comparison, we introduce the results obtained using two alternative fee structures. Furthermore, we conduct a sensitivity analysis encompassing diverse parameter configurations.

2. Valuation of fair fees

2.1. HWM fee structure and investment account dynamics

In this section, we utilize a risk-neutral pricing approach to determine fair insurance fees for the GMWB under the HWM fee structure. As the initiation of a typical VA contract embedded with GMWB, the PH pays an initial premium (denoted as P) and invests this premium in a predetermined portfolio of funds. The insurer establishes an investment account F to monitor the performance of the policy fund with $F_0 = P$, where $\{F_t\}_{0\leq t\leq T}$ represent the dynamic of the investment account F until the maturity date T.

Following the conventions of option pricing literature, we denote S_t as the dynamics of the assets underlying the VA policy, driven by a geometric Brownian motion. We assume that the existence of a risk-neutral probability measure Q such that all discounted asset price processes are Q-martingales. Under the risk-neutral probability measure Q, the evolution of S_t is presumed to follow the subsequent stochastic differential equation (SDE),

$$\mathrm{d}S_t = rS_t\mathrm{d}t + \sigma S_t \mathrm{d}B_t^Q, \tag{2.1}$$

where B_t^Q follows a standard Brownian motion. The parameter r denotes the continuously compounded risk-free interest rate and σ denotes the volatility of the investment return.

In our modeling framework, we adopt a static approach, wherein the policyholder is assured the ability to withdraw a constant value w per time unit. This guarantee remains in force until the entire initial premium has been fully reimbursed, regardless of the performance of the investment account F. In other words,the GMWB matures at $T = F_0/w$.

In the scenario of the HWM fee structure, we introduce two parameters for the insurance fees, including the constant fee rate c and the HWM fee rate α. The constant fee rate c is applied as a proportion of the present value of the investment account F_t when the account value falls below a predetermined threshold θ. Conversely, when the account value surpasses this fixed threshold θ and achieves a new peak (the high-water mark, HWN), the HWM fee rate α is levied as a proportion of the positive change from the previous highest position. Therefore, the dynamics of the investment account F_t, depleted by the constant continuous static withdrawal w and the continuous flow of fee charges determined by these two components, can be expressed as follows:

$$\mathrm{d}F_t = (r - c1_{\{F_t\leq\theta\}})F_t\mathrm{d}t + \sigma F_t \mathrm{d}B_t^Q - \alpha 1_{\{F_t>\theta\}}\mathrm{d}M_t - w\mathrm{d}t, \tag{2.2}$$

where

$$M_t := \sup_{0\leq s\leq t}\{F_s\} \tag{2.3}$$

denotes the HWM of the investment account at time t. Note that this stochastic differential equation is applicable only when $F_t > 0$. Once the investment account value hits 0, it remains at 0 until the maturity date.

The HWM fee structure can be viewed as a more encompassing framework compared to two other fee structures. It aligns with the rationale that insurers charge higher insurance fees for superior returns in the policy fund and lower fees for lower returns, promoting efficient management and enhanced performance. For the sake of comparison, we outline the two alternative fee structures as follows:

If the HWM fee α is set to 0, we obtain a state-dependent fee structure:

$$\mathrm{d}F_t = (r - c1_{\{F_t\leq\theta\}})F_t\mathrm{d}t + \sigma F_t \mathrm{d}B_t^Q - w\mathrm{d}t. \tag{2.4}$$

If the threshold $\theta \to \infty$, we obtain a static fee structure:

$$\mathrm{d}F_t = (r - c)F_t\mathrm{d}t + \sigma F_t \mathrm{d}B_t^Q - w\mathrm{d}t. \tag{2.5}$$

The conventional constant fee structure is deemed unfavorable from a risk management standpoint, and it can lead to a misalignment of fee income and option value from the insurers' perspective (see Bernard et al., 2014). The fixed fee rate encourages policyholders to surrender their contracts, as they can lapse the existing contract and enter into a new policy fund repeatedly when the old guarantees are depleted. Conversely, when fund performance is poor, the fees charged by insurers decrease proportionally. Conversely, in times of strong fund performance, fee income increases while the option value decreases. To address these issues, the state-dependent fee structure correlates the fee rate with the evolving investment account value. However, this characteristic may cause insurers to deliberately prolong the fee-charging process. In comparison, the HWM fee structure appears to be a more reasonable mechanism.

We denote τ as the stopping time satisfying $\tau := inf\{t : F_t \leq 0\}$, a crucial point for our subsequent analysis. In the forthcoming subsection, we formulate pricing equations from the insurer's standpoint. These results will enable us to define and compute two risk measures essential for managing the risk associated with the GMWB.

2.2. Risk-neutral pricing

We can derive the pricing of the GMWB from the insurer's perspective by ensuring the insurer's liability matches its income. The insurer incurs no financial obligation until the account value reaches zero, as both withdrawals and the refunded balance at maturity are sourced from the account itself. The insurer's cost arises from the guaranteed payments after the investment fund is depleted before the maturity date. Hence, the present value of the cost (gross liability) for an insurer offering the GMWB rider is given by,

$$w\int_{\tau}^{T} e^{-rt}dt 1_{\{\tau<T\}} = \frac{w}{r}(e^{-r\tau} - e^{-rT})1_{\{\tau<T\}}. \tag{2.6}$$

To compensate for the guarantees, the insurer receives an allocation of the total fee (c,α) which is a component of the total fee (c_g,α_g). Typically, fees determined from the policyholders' perspective are significantly higher, as only a portion of these fees are allocated to fund the GMWB rider, given the existence of other expenses. The income of the insurer encompasses the accumulated fee charges from the inception of the contract to the earlier of the stopping time τ and maturity date T. This income consists of two integrals, encompassing both the constant component and the HWM component. Therefore, the present value of the accumulated fee income is given by,

$$c\int_{0}^{\tau\wedge T} e^{-rt}F_t 1_{\{F_t<\theta\}}dt + \alpha\int_{0}^{\tau\wedge T} e^{-rt}1_{\{F_t\geq\theta\}}dM_t. \tag{2.7}$$

To equate the gross liability to the income, we employ no-arbitrage methods to ascertain the fair fees (c,α) by

$$\frac{w}{r}E^Q\left[(e^{-r\tau} - e^{-rT})1_{\{\tau<T\}}\right] = E^Q\left[c_w\int_{0}^{\tau\wedge T} e^{-rt}F_t 1_{\{F_t<\theta\}}dt + \alpha_w\int_{0}^{\tau\wedge T} e^{-rt}1_{\{F_t\geq\theta\}}dM_t\right]. \tag{2.8}$$

In forthcoming section 3, we further investigate the perspective of the insurer, emphasizing the computation of tail probabilities and expectations concerning the net liability.

3. Risk management

3.1. Net liability

In Section 2, we derived the gross liability, quantifying the insurer's cost in holding the GMWB rider with an HWM fee structure. Distinct from other financial instruments, the fees

levied by the insurer are linked to the investment account, thus intertwining the risk with the income aspect from the insurer's standpoint. In the subsequent analysis, we develop the concept of net liability, defined as the gross liability subtracted from the accumulated income. Unlike the previous model, this time we construct both the gross and net liability under a physical probability measure P. Suppose that the dynamics of the underlying policy fund S_t follows a geometric Brownian motion,

$$\mathrm{d}S_t = \mu S_t \mathrm{d}t + \sigma S_t \mathrm{d}B_t, \tag{3.1}$$

where $\{B_t\}_{0 \leq t \leq T}$ is a standard Brownian motion under the physical probability measure P. The parameter μ denotes the expected return of the investment account and σ represents the volatility of the investment account.

The dynamics of the investment account are described by the following stochastic differential equation:

$$\mathrm{d}F_t = (r - c_g 1_{\{F_t \leq \theta\}}) F_t \mathrm{d}t + \sigma F_t \mathrm{d}B_t - \alpha_g 1_{\{F_t \geq \theta\}} \mathrm{d}M_t - w \mathrm{d}t, \tag{3.2}$$

where the fees (c_g, α_g) are charged based on the HWM threshold θ, as elaborated in the previous section.

Utilizing the aforementioned equations, we can deduce the net liability by computing the difference between the gross liability and the income. Note that the fee pair used in the income calculation is denoted as (c_w, α_w). We represent the present values of the net liability for GMWB as:

$$L = \frac{w}{r}(e^{-r\tau} - e^{-rT}) 1_{\{\tau < T\}} - c_w \int_0^{\tau \wedge T} e^{-rt} F_t 1_{\{F_t < \theta\}} dt - \alpha_w \int_0^{\tau \wedge T} e^{-rt} 1_{\{F_t \geq \theta\}} dM_t. \tag{3.3}$$

In most scenarios, a negative net liability is anticipated, indicating a profitable product. However, for risk management purposes, our focus shifts to instances with positive net liabilities. In the subsequent analysis, we delve into the upper-tail distribution of the net liability using two risk measures.

3.2. Risk measures

The fundamental approach to risk management in this paper revolves around reserving adequate capital to cover potential losses in adverse scenarios. Obtaining the distribution of the net liability, especially under the intricate HWM fee structure, proves to be challenging. By calculating specific risk measures, we can approximate the extreme distribution of the net liability, aiding in the determination of effective risk strategies. The two widely utilized risk measures are the Value-at-Risk (VaR) and the Conditional-Tail-Expectation (CTE). The quantile risk measure VaR is defined as:

$$VaR_\alpha := inf\{y : P(L \leq y) \geq \alpha\}, 0 \leq \alpha \leq 1. \tag{3.4}$$

The quantile risk measure, VaR, is interpreted as the minimum capital necessary to ensure that there is sufficient funding to cover future liabilities with a probability of at least α. In our framework, the net liability L is modeled as a continuous random variable, allowing the VaR to be simplified as:

$$P(L > VaR_\alpha) = 1 - \alpha. \tag{3.5}$$

The conditional tail expectation risk measure, CTE, is defined as,

$$CTE_\alpha := E[L | L > VaR_\alpha]. \tag{3.6}$$

This translates to the minimum capital needed to cover the average loss in scenarios where the net liabilities surpass the quantile risk measure VaR, with a probability of at most $1-\alpha$. By incorporating the risk-neutral pricing outcomes from the insurer's standpoint, as presented in Section 2, we simulate the probability density function of the net liability and estimate the risk measures using order statistics, in the following section.

4. Numerical illustration

In this section, we present numerical examples to achieve two main objectives. Firstly, we numerically solve the stochastic differential equations, as outlined in Section 2, to determine the fair fees of VAs with the embedded GMWB rider under diverse conditions from the insurer's viewpoint. Secondly, we compute the quantile risk measure VaR and conditional tail expectation CTE within the same framework as the initial work. We conduct a comparative analysis between the traditional static fee structure and the HWM fee structure. Subsequently, we assess the sensitivity of the fair fees and risk measures to various parameters. Finally, we ascertain the optimal surrender time and its corresponding policy value.

4.1. Simulation setting and sensitive analysis

The Monte Carlo method proves advantageous for simulating the paths of underlying stochastic processes that describe asset dynamics and the numerical simulation in this section follows the subsequent approach. For each given pair of fair insurance fees (c,α), we generate N sample paths. For each sample path, we generate account values with a time step of Δt from time 0 to the earlier of maturity $T=\frac{F_0}{w}$ and the first instance when the account is depleted. We compute the average of the N no-arbitrage values representing the expectation under the risk-neutral measure Q. This enables us to establish the relationships between the fair insurance fees and the no-arbitrage value, aiding in determining a suitable combination of the fees while fixing one parameter to (2.8). For comparative purposes, we vary the risk-fee interest rate r and the volatility of the investment account σ to discern changing laws. Once the fair fees are determined, the calculation of risk measures is performed based on various parameters and their corresponding fee pairs. Likewise, we conduct a sensitivity analysis to understand the impact of different conditions on the two risk measures.

Table 1. Parameter inputs

Description	Parameter	Base case	Sensitivity analysis
Initial premium	F_0	100	–
Withdrawal per time unit	w	4	2,3,5
The threshold for the constant and HWM fee	θ	120	–
Interest rate	r	2%	1%,3%
Expected return of the investment account	μ	8%	6%,10%
Volatility	σ	20%	15%
HWM fee	α	0.3	0,0.1,0.5

To determine the pair of fair insurance fees (c,α) under the HWM fee structure, we adopt an approach where we either fix the HWM fee rate $\alpha\in[0,1]$ and derive the state-dependent constant fee rate $c$within the same range, or vice versa. The parameters used in this determination are specified in Table 1.

To price the GMWB from the insurer's perspective, we focus on a scenario where the withdrawal rate w is set to 4 per time unit, the initial premium F_0 is set to 100, and the

maturity date T is 25. For our calculations, we set the time interval as $\Delta t = 0.01$ and the number of paths as $N = 1000$.

Figure 1 illustrates the variations in gross liability and income of insurers in relation to the selection of the constant fee rate c under different α, with the base case $r = 0.02$ and $\sigma = 0.20$. It is evident that both gross liability and income increase with an increase in c for a fixed α. Each intersection of the two curves, belonging to the same color group, represents a solution for c. Notably, c tends to be higher with lower α within the given basic setting r and σ.

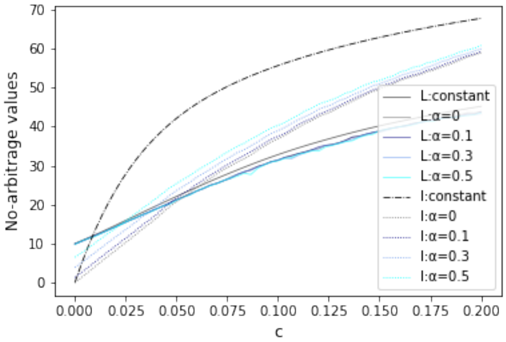

Fig. 1. Relationship between c and the no-arbitrage value of Liability and Income

To identify the factors influencing the value of fair fees, we employ the binary search method to determine the constant fee c for each pair of parameters. Table 2 presents the results of this search for various risk-free interest rates r ranging from 1% to 3%, and the volatility of the investment account σ ranging from 15% to 20%. The fees presented in Table 2 represent the total fees (c,α).

Table 2. Fair constant fees c under different parameters

HWM fee	$r = 1\%$		$r = 2\%$		$r = 3\%$	
	$\sigma = 0.15$	$\sigma = 0.2$	$\sigma = 0.15$	$\sigma = 0.2$	$\sigma = 0.15$	$\sigma = 0.2$
Constant	0.01172	0.01563	0.00586	0.00781	0.00195	0.00488
$\alpha = 0(SD)$	0.04043	0.12187	0.01699	0.05449	0.00820	0.02929
$\alpha = 0.1$	0.03750	0.11719	0.01406	0.04688	0.00527	0.02285
$\alpha = 0.3$	0.03047	0.09844	0.00762	0.03633	0.00000	0.01055
$\alpha = 0.5$	0.02168	0.07449	0.00117	0.02051	0.00000	0.00000

In Table 2, "Constant" corresponds to the constant fee structure, and "SD" corresponds to the state-dependent fee structure (the special case $\alpha = 0$), while we consider three other levels of HWM fee rate α, namely 0.1, 0.3 and 0.5. For the HWM fee structures, the trends are clear and distinct.

Firstly, we observe that the constant fee rate c generally decreases as HWM fee α increases for all three risk-free rates r and two different volatility σ. However, this impact is marginal and even approaches zero with a high risk-free interest rate $r = 3\%$ and $\alpha = 0.3, 0.5$, which can be attributed to the fee structure itself. Turning our attention to the risk-free interest rate r, we note that for a fixed HWM fee rate α and volatility σ, the constant fee rate c tends to decrease with a higher interest rate r. This relationship is inherent in equation (2.8) since

a high interest rate leads to a larger dynamic account by reducing the fee charges. Lastly, the constant fee rate c significantly increases with an increase in volatility σ. This aligns with our traditional intuition that more fee charges are required for more volatile investments. Nevertheless, the patterns for the constant fee structure remain uncertain. Generally, the fees under a constant fee structure are notably lower than those under the other fee structures, likely due to the unconditional fee charge.

Next, we calculate two risk measures based on the risk-neutral fees derived earlier. In addition to the parameters used previously, we incorporate the expected return of the investment accountμ into the policy fund dynamics, ranging from 6% to 10%. The recommended risk measures for determining additional asset requirements for equity-linking are $VaR_{0.95}$ and $CTE_{0.95}$. The risk measure $VaR_{0.95}$ is estimated using the order statistic $L_{0.95N}$ and the $CTE_{0.95}$ is estimated using the arithmetic average of the largest $0.05N$ order statistics. Through Monte Carlo simulation, we construct the density function of the net liability L and investigate the impact of various factors on it.

Finally, we conduct sensitivity tests on each parameter involved in the computation of risk measures while keeping all other factors consistent with the valuation basis.

The impact that the withdrawal rate w can influence the distribution of net liability can be comprehended from two perspectives from the policyholder's standpoint. On the one hand, the GMWB itself can be perceived as a complex 'put option'. More withdrawals translate to greater realized benefits from the GMWB. On the other hand, as the policyholder can receive the remaining account balance at maturity, the GMWB can also be seen as a 'call option' (see Feng 2017). Consequently, the policyholder is incentivized to maintain a sufficiently low withdrawal rate to ensure that the account remains positive, thus obtaining the benefit of equity returns at maturity. Table 3 shows the effect of w on the risk measures of net liability under the basic case levels of other parameters within the HWM fee structure.

Table 3. Impact of withdrawal rate on risk measures of L

w	2	3	4	5
$VaR_{0.95}$	-7.11707	3.92735	8.46264	12.31166
$CTE_{0.95}$	7.82734	16.22349	20.66039	22.30615

In the numerical examples provided above, we notice that both $Var_{0.95}$ and $CTE_{0.95}$ increase with higher withdrawal rates w. This phenomenon can be explained as follows: with a higher withdrawal rate, the investment account decreases, leading to reduced fee income. Simultaneously, the gross liability is higher due to an earlier stopping date τ and more frequent withdrawals. Consequently, the density function of the net liability shifts to the right, resulting in larger values for upper-tail measures.

Figure 2 illustrates the density function of net liability under the basic parameter settings with varying withdrawal rates w. A notable observation is that the distribution becomes more concentrated as w This is quantified by a reduction in standard deviations from 159.64 to 34.73 as the withdrawal rate w ranges from 2 to 5. Additionally, we observe that net liabilities are predominantly negative in most scenarios, indicating that insurers generate profits from these contracts. The reasons for this observation are elaborated from two aspects in Feng and Vecer (2017).

Figure 3 displays the density functions for different fee structures. A notable observation is that the net liability under the constant fee structure is significantly larger than that of the HWM fee structure, confirming our earlier judgment that the HWM fee structure is more advantageous for sellers. Next, we examine the relationships between the distribution

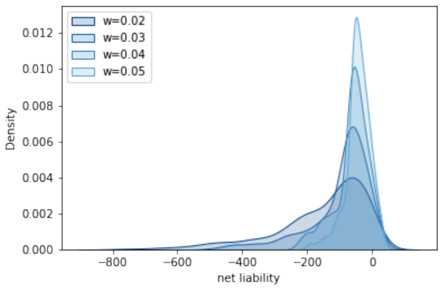

Fig. 2. Density function with various w

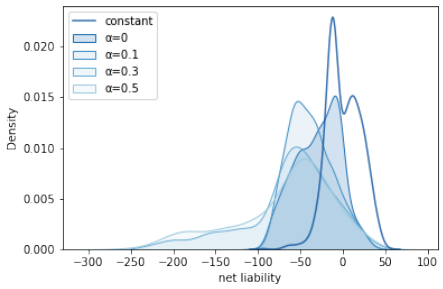

Fig. 3. Density function with various α

of net liability and the HWM fee rate α to select parameters for the HWM fee structure. In contrast to Figure.3, the concentrations of the density function increase from 31.89 to 67.68 as α increases from 0 to 0.5. The curve also shifts to the left with increasing α. Table 4 presents the values of $VaR_{0.95}$ and $CTE_{0.95}$ under different α along with the corresponding constant fee rate c while keeping the other basic parameters consistent.

Table 4. Impact of fee structures on risk measures of L

Fee structures	Constant	$\alpha = 0(SD)$	$\alpha = 0.1$	$\alpha = 0.3$	$\alpha = 0.5$
$VAR_{0.95}$	33.59156	10.39162	9.50535	8.46264	8.16489
$CTE_{0.95}$	38.73753	19.83707	20.22424	20.66039	18.47178

We observe that the risk measures increase as α becomes higher.To provide reasonable explanations, we calculate the gross liability and the income under different α with matching c values. We find that the differences in gross liabilities for various α are insignificant, whereas the incomes noticeably decrease with increasing α. This reduction in income can be attributed to the fee structure. Analyzing the pricing results, we observe that higher α values lead to a decrease in the constant fee rate c. Therefore, the direction of income change depends on the balance between the constant fee part and the HWM fee part. In most scenarios, the constant fee part holds more weight, aligning with our numerical results and contributing to the observed tendency.

We view VaR_q as a function of q and present the function in Fig.4 for both the constant and the HWM fee structure, using the basic parameter set.

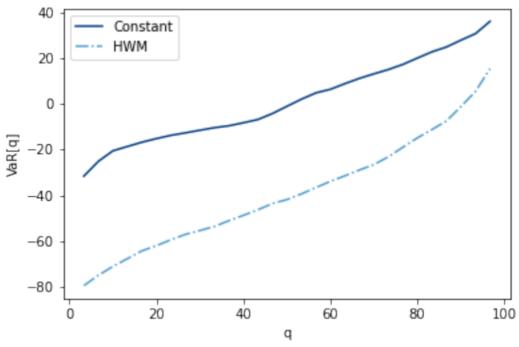

Fig. 4. Function $VaR[q]$ for different fee structure

As mentioned earlier, under the same conditions, $VaR_{Constant}[q] > VaR_{HWM}[q]$ consistently. From the insurer's standpoint, the novel fee structure reduces the net liability, thereby mitigating the risk.

Table 5 presents the risk measures under varying levels of the expected return of the investment account μ, the risk-free interest rate r and the volatility of the investment account σ.

Table 5. Sensitivity analysis of risk measures for the GMWB rider

μ	0.06	0.08	0.10
$VAR_{0.95}$	16.60761	8.46264	-0.66253
$CTE_{0.95}$	26.21322	20.66039	13.83689
σ	0.15	0.20	0.25
$VAR_{0.95}$	-7.57755	8.46264	17.94125
$CTE_{0.95}$	6.11116	20.66039	26.20981
r	0.01	0.02	0.3
$VAR_{0.9}$	28.89721	20.68489	11.32942
$CTE_{0.9}$	35.13599	29.08121	21.16528

We first exam the impact of the expected return and the volatility on the tail distribution, keeping all other parameters as given in the base case. When μ is higher, the income increases and the gross liability shrinks, leading to lower tail risk measures. In a high volatility market, insurers bear heavier liabilities. Next, we investigate how the risk-free interest rate r affects the results. When r decreases from the base case, the net liabilities increase. Not surprisingly, with a lower risk-free interest rate, "savings instruments" such as annuities and variable annuities become less appealing to investors, making these products less advantageous for insurers

4.2. Surrender behaviour analysis

Considering surrender behaviors, we calculate the optimal surrender time and the corresponding policy value embedded with GMWB using the Least Squares Monte Carlo Simulation (LSMC) based on the previously calculated fees.

First, we introduce the fundamentals of LSMC and its adaptation for policy valuation. This method was originally developed for pricing American options. In this process, we generate N sample paths of the underlying asset value. Then, by iteratively solving regression equations starting from the maturity date, we obtain the optimal surrender execution time and the corresponding payoff for each sample path. Finally, we take the average of all N discounted payoffs as the simulated value. In our paper,

- t_*^i represents the optimal surrender time for the i-th path.
- V_t^i signifies the policy value of the i-th path at time t, calculated as the maximum of the surrender value and the holding value.
- C_t^i refers to the continuously holding value given the current account value F_t^i, defined as $e^{-rt}E(V_{t+1}^i|F_t^i)$.
- L_t^i denotes the surrendering value.

- p is the penalty rate designed to discourage surrender behaviors.

Further details are provided below:

1) Generate N paths of the account value F_t^i using Monte Carlo simulation in a similar manner as in the previous section. Set the initial optimal surrender time $t_*^i = T$ and the payoff at maturity date is $V_T^i = e^{-rT} F_T^i 1_{\{\tau^i > T\})} + \frac{w}{r}(1 - e^{-rT})$

2) t=T-1,...,2,1,

   - Regress V_{t+1}^i on F_t^i to obtain $E(V_{t+1}^i | F_t^i) = f(F_t^i)$. Then $C_t^i = e^{-rt} E(V_{t+1}^i | F_t^i) + e^{-rt} w$. $L_t^i = (1-p) F_t$.
   - If $C_t^i < L_t^i$, surrender is a better choice, in which case, reset t_*^i as t. Otherwise, the policyholder will choose to continuously hold the policy, with t_*^i remaining unchanged. The policy value at t is $V_t^i = max(C_t^i, L_t^i)$.
   - Repeat the above steps from T-1 to 1, and we can obtain the final t_*^i as the optimal surrender time for the i-th sample path. At this point, for any path i, the surrender policy is known. So we should find the $V_{t_*^i}$ as the optimal policy value of this path.

3) The price of this policy is the average of the discounted optimal policy value of all the N paths. That is,

$$\hat{V_0} = \frac{1}{n} \sum_{i=1}^{n} e^{-rt_*^i} V_{t_*}^i. \tag{4.1}$$

Under the basic set, we can roughly sketch out the density of the optimal surrender time with N sample paths. Since our purpose is to discourage early surrender behaviors, the penalty should be dynamic, decreasing with time until zero. We set the dynamic charge rate as:

$$p_t = (1 - 0.04 * t) p_0, \tag{4.2}$$

where p_0 is the maximum rate during the policy.

For instance, when the p_0 is 0.1,the dynamic surrendering charge is :

Table 6. Dynamic surrender charge rate

year	0	5	10	15	20	25
charge rate	0.10	0.08	0.06	0.04	0.02	0.00

Fig.5 gives the density function of the optimal time t_*. Most t_* fall in [20,25]. Corresponding to it, the distribution of the V_{t_*} is shown in Fig.6. The probability density ranks high around the initial premium.

For different penalty rates, the results may vary. Considering the p ranges from 0 to 0.8, the change of t_* and the V_{t_*} are provided as follows.

From the observations in Figure 7, it is evident that optimal surrender behavior diminishes as the penalty rate $p$increases. This substantiates the efficacy of a dynamic penalty in deterring premature surrendering. Notably, when the penalty rate exceeds 0.15, it is optimal to retain the policy until maturity. Moreover, the average policy value consistently remains below 100 when surrender incurs a penalty.

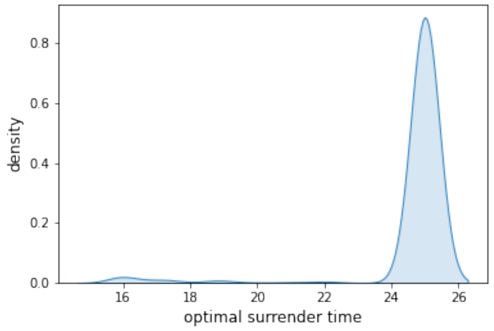

Fig. 5. Density function of t_*

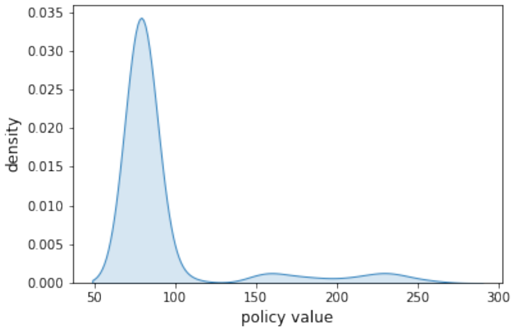

Fig. 6. Density function of V_{t_*}

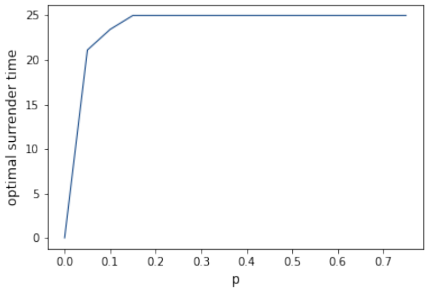

Fig. 7. Density function with various w

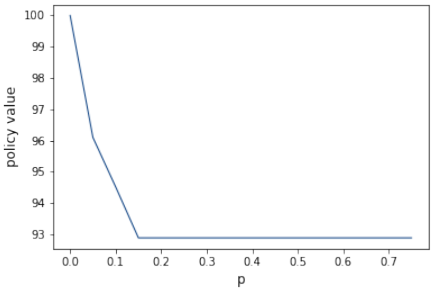

Fig. 8. Density function with various α

5. Conclusion and future works

This study extends the HWM fee structure to encompass withdrawal-based guarantees. Initially, we determine equitable fee pairs for GMWB with the HWM fee structure utilizing the traditional risk-neutral pricing framework. Subsequently, we delve into risk strategies from a reserving perspective.

However, it's important to note that the Monte Carlo method used can be computationally intensive and may introduce biases in simulations due to errors. Future research should consider alternative analytical methods or algorithms to mitigate these computational challenges and reduce errors in estimating risk measures associated with rare events. Furthermore, incorporating policyholders' behaviors into the analysis would enhance the comprehensiveness of the model. Since our current model is tailored for a single contract, an avenue for further exploration involves extending the analysis to a diverse array of contracts belonging to various categories. Additionally, integrating appropriate hedging instruments to effectively manage the risk associated with VAs is an important avenue for future research.

References

[1] Anna Rita Bacinello, Pietro Millossovich, Annamaria Olivieri, and Ermanno Pitacco. Variable annuities: A unifying valuation approach. Insurance: Mathematics and Economics, 49(3):285–297, 2011.

[2] Runhuan Feng, Guojun Gan, and Ning Zhang. Variable annuity pricing, valuation, and risk management: a survey. Scandinavian Actuarial Journal, 2022(10):867–900, 2022.

[3] Laura Ballotta, Ernst Eberlein, Thorsten Schmidt, and Raghid Zeineddine. Variable annuities in a lévy-based hybrid model with surrender risk. Quantitative Finance, 20(5):867–886, 2020.

[4] Juan Luo, Shuxin Wang, Lulu Dan, Rui Zhang, and Liang Bi. Research on the influence of payment methods on the control of medical insurance expenses—based on empirical analysis of double difference. Frontiers in Public Health, 10:938831, 2022.

[5] Dongliang Yang, Zhichao Ren, and Ge Zheng. The impact of pension insurance types on the health of older adults in china: a study based on the 2018 charls data. Frontiers in Public Health, 11:1180024, 2023.

[6] Hyukjun Gweon and Shu Li. A hybrid data mining framework for variable annuity portfolio valuation. ASTIN Bulletin: The Journal of the IAA, 53(3):580–595, 2023.

[7] X Sheldon Lin and Shuai Yang. Fast and efficient nested simulation for large variable annuity portfolios: A surrogate modeling approach. Insurance: Mathematics and Economics, 91:85–103, 2020.

[8] Moshe A Milevsky and Thomas S Salisbury. Financial valuation of guaranteed minimum withdrawal benefits. Insurance: Mathematics and Economics, 38(1):21–38, 2006.

[9] Runhuan Feng and Hans W Volkmer. An identity of hitting times and its application to the valuation of guaranteed minimum withdrawal benefit. Mathematics and Financial Economics, 10:127–149, 2016.

[10] Jingjiang Peng, Kwai Sun Leung, and Yue Kuen Kwok. Pricing guaranteed minimum withdrawal benefits under stochastic interest rates. Quantitative Finance, 12(6):933–941, 2012.

[11] Min Dai, Yue Kuen Kwok, and Jianping Zong. Guaranteed minimum withdrawal benefit in variable annuities. Mathematical Finance: An International Journal of Mathematics, Statistics and Financial Economics, 18(4):595–611, 2008.

[12] Alexander Kling, Frederik Ruez, and Jochen Russ. The impact of stochastic volatility on pricing, hedging, and hedge efficiency of withdrawal benefit guarantees in variable annuities. ASTIN Bulletin: The Journal of the IAA, 41(2):511–545, 2011.

[13] Ludovic Goudenège, Andrea Molent, and Antonino Zanette. Gaussian process regression for pricing variable annuities with stochastic volatility and interest rate. Decisions in Economics and Finance, 44:57–72, 2021.

[14] Claudio Fontana and Francesco Rotondi. Valuation of general gmwb annuities in a low interest rate environment. Insurance: Mathematics and Economics, 112:142–167, 2023.

[15] Carole Bernard and Thorsten Moenig. Where less is more: Reducing variable annuity fees to benefit policyholder and insurer. Journal of Risk and Insurance, 86(3):761–782, 2019.

[16] Carole Bernard, Mary Hardy, and Anne MacKay. State-dependent fees for variable annuity guarantees. ASTIN Bulletin: The Journal of the IAA, 44(3):559–585, 2014.

[17] Zhenyu Cui, Runhuan Feng, and Anne MacKay. Variable annuities with vix-linked fee structure under a heston-type stochastic volatility model. North American Actuarial Journal, 21(3):458–483, 2017.

[18] David Landriault, Bin Li, Dongchen Li, and Yumin Wang. High-water mark fee structure in variable annuities. Journal of Risk and Insurance, 88(4):1057–1094, 2021.

[19] Gu Wang and Bin Zou. Optimal fee structure of variable annuities. Insurance: Mathematics and Economics, 101:587–601, 2021.

[20] J Lars Kirkby and Jean-Philippe Aguilar. Valuation and optimal surrender of variable annuities with guaranteed minimum benefits and periodic fees. Scandinavian Actuarial Journal, 2023(6):624–654, 2023.

[21] Runhuan Feng and Bingji Yi. Quantitative modeling of risk management strategies: Stochastic reserving and hedging of variable annuity guaranteed benefits. Insurance: Mathematics and Economics, 85:60–73, 2019.

[22] Frédéric Godin, Emmanuel Hamel, Patrice Gaillardetz, and Edwin Hon-Man Ng. Risk allocation through shapley decompositions, with applications to variable annuities. ASTIN Bulletin: The Journal of the IAA, 53(2):311–331, 2023.
