# Supplementary figures and images for: Guaranteed minimum withdrawal benefits with high-water mark fee structure
Source: PLoS One. 2024 May 21;19(5):e0302740. doi: 10.1371/journal.pone.0302740 (PMC11108128; doi:10.1371/journal.pone.0302740)

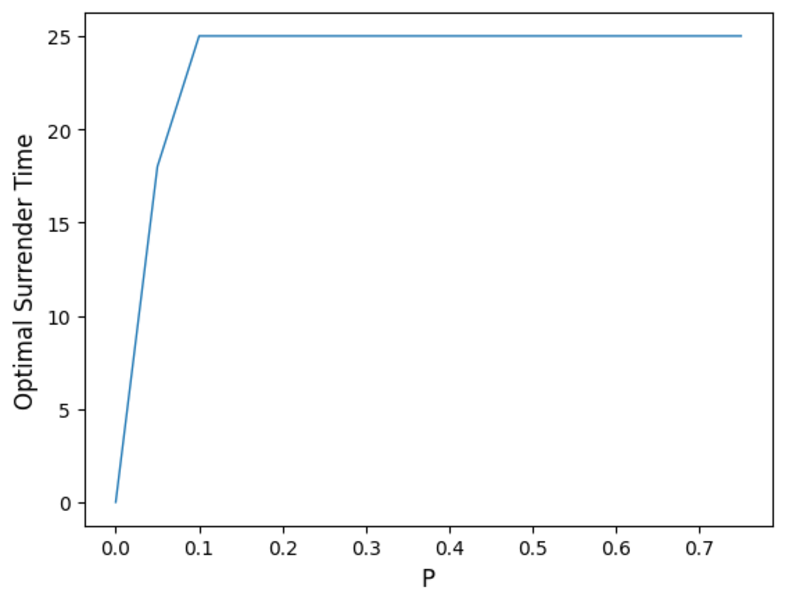

Supplement: S1 File — (ZIP) [file pone.0302740.s002.zip › Highwatermark (jump regime switch)/33.png]

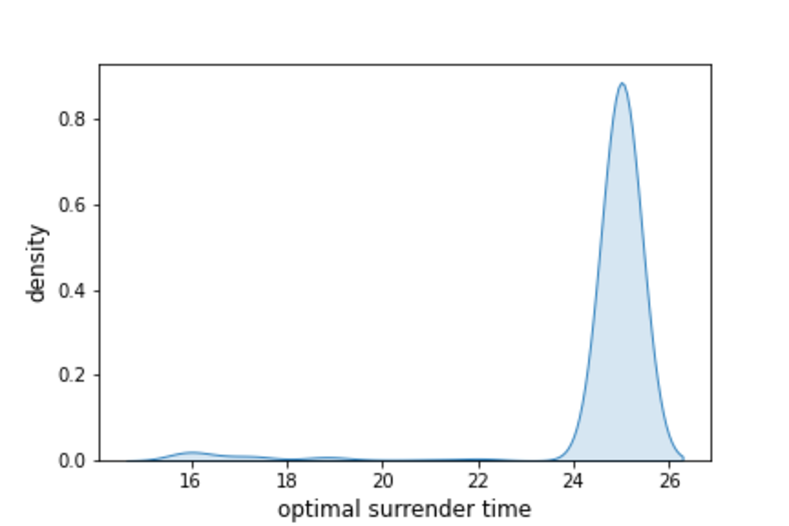

Supplement: S1 File — (ZIP) [file pone.0302740.s002.zip › Highwatermark (jump regime switch)/444.png]

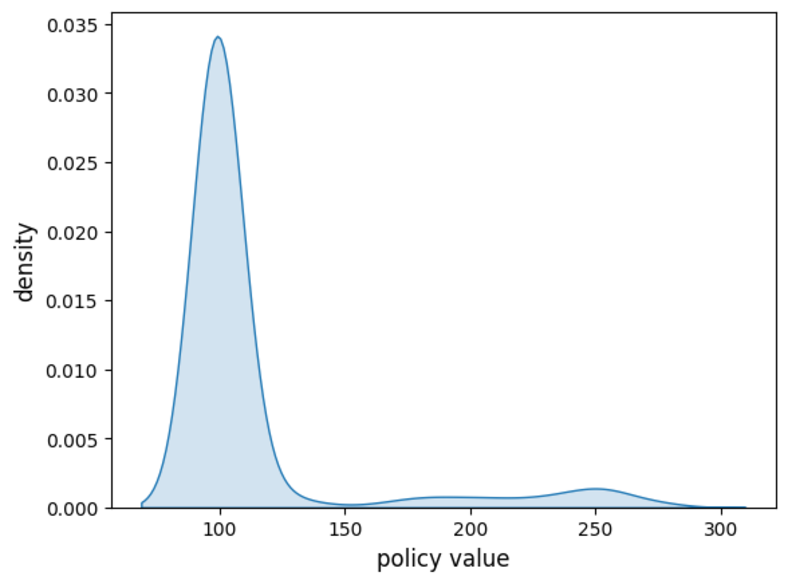

Supplement: S1 File — (ZIP) [file pone.0302740.s002.zip › Highwatermark (jump regime switch)/555.png]

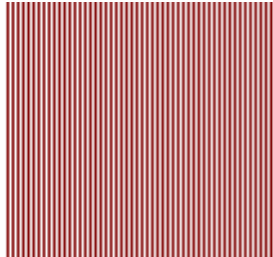

(a)

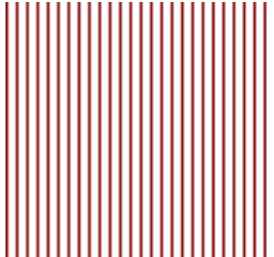

(b)

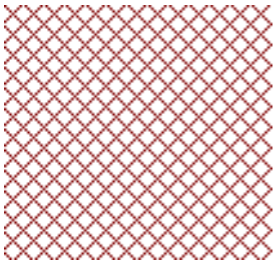

(c)

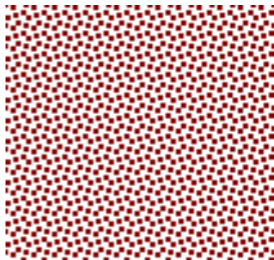

(d)

Supplement: S1 File — (ZIP) [file pone.0302740.s002.zip › Highwatermark (jump regime switch)/OT10000F1.pdf]

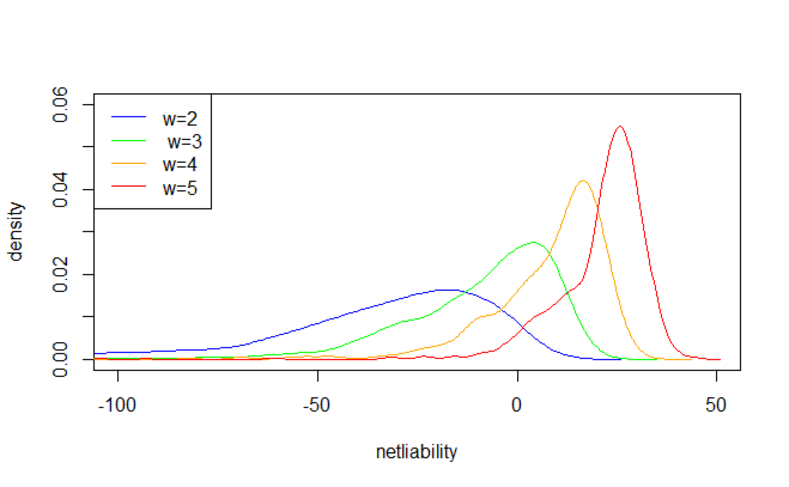

Supplement: S1 File — (ZIP) [file pone.0302740.s002.zip › Highwatermark (jump regime switch)/netliability.png]

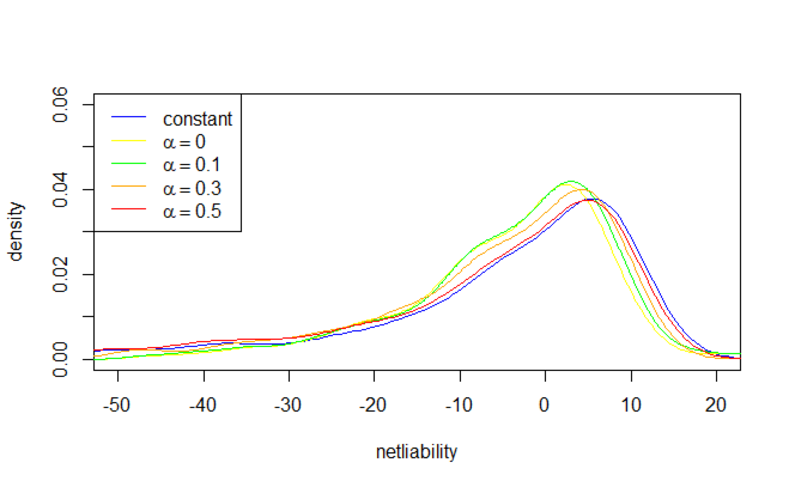

Supplement: S1 File — (ZIP) [file pone.0302740.s002.zip › Highwatermark (jump regime switch)/netliabilityalpha.png]

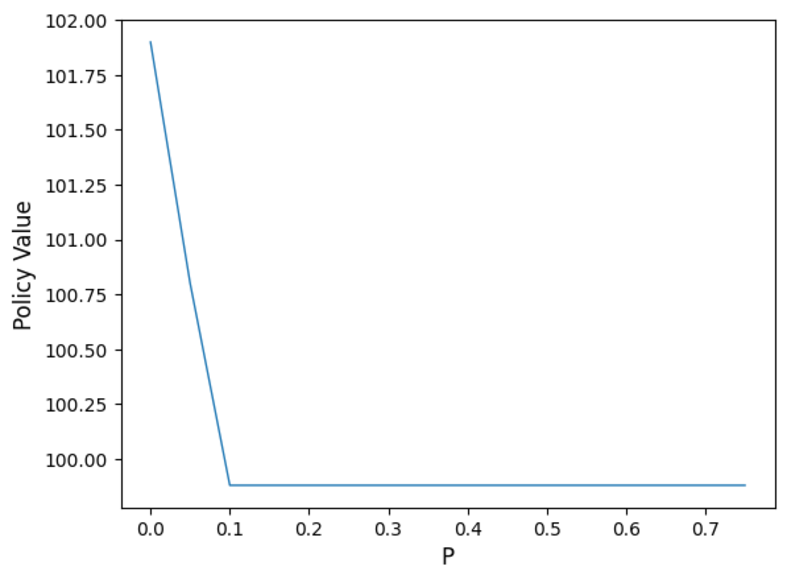

Supplement: S1 File — (ZIP) [file pone.0302740.s002.zip › Highwatermark (jump regime switch)/p_policyvalue.png]

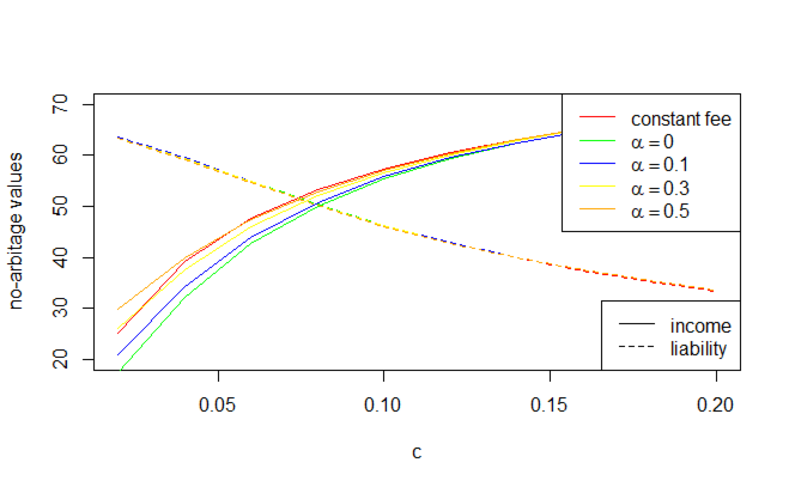

Supplement: S1 File — (ZIP) [file pone.0302740.s002.zip › Highwatermark (jump regime switch)/pricec.png]
